# Deep Vein Thrombosis in the Humeral Vein After Implantable Cardioverter-Defibrillator Implantation: A Family Physician's Perspective

**DOI:** 10.7759/cureus.50827

**Published:** 2023-12-20

**Authors:** Pedro Alves, João Silva, João Ribeiro, Sónia Moreira

**Affiliations:** 1 Family Medicine, USF (Unidade de Saude Familiar) Tâmega, Administração Regional de Saúde do Norte, Porto, PRT; 2 Family Medicine, USF (Unidade de Saude Familiar) Alpendorada, Administração Regional de Saúde do Norte, Porto, PRT

**Keywords:** tako-tsubo cardiomyopathy (ttc), humeral vein, implantable cardioverter-defibrillators, subclavian vein thrombosis, upper extremity deep venous thrombosis

## Abstract

Upper extremity deep vein thrombosis (DVT) is an uncommon, under-reported, and difficult-to-diagnose condition. Although the strong provoking risk factors of venous thromboembolism are well described in the literature, the majority of cases are provoked by weak risk factors or are even considered unprovoked. In this case report, we describe a rare case of a brachial DVT in a woman in her 40s following implantable cardioverter-defibrillator (ICD) implantation. In her first evaluation, slight left arm edema and brachialgia were noted, and physiotherapy was prescribed. One month later, the patient was reevaluated because her complaints did not resolve, and an upper extremity venous ultrasound was done to exclude complications due to ICD implantation. The ultrasound identified an old DVT, which had been completely recanalized. The patient was then referred to a vascular surgery specialty consultation, which confirmed the diagnosis, and an anticoagulant was prescribed for three months. The symptoms resolved, and the patient did not report any more pain.

## Introduction

Venous thromboembolism (VT), which includes both deep vein thrombosis (DVT) and pulmonary embolism [1], affects nearly 10 million people worldwide each year [2]. Unlike men, women have a higher risk of VT between the ages of 20 and 40 years due to exposure to reproductive risk factors [3].

VT is a multifactorial disease that can be triggered by one or many risk factors, which can act synergistically or additively [1]. Strong provoking risk factors for VT include active cancer, antiphospholipid syndrome, major physical trauma, prolonged immobility, surgery lasting longer than 30 minutes, and hospitalization for acute illnesses [1]. However, the majority of VT cases are triggered by weak risk factors or are even considered unprovoked [4]. There are many such weak risk factors, including pacemaker implantation and implantable cardioverter-defibrillators (ICDs) [1]. ICDs represent a growing technique used both in primary and secondary prevention of cardiac arrest [5]. Despite being an infrequent cause of VT [6], many cases have already been reported in the literature.

In this report, we describe a rare case of a brachial DVT following ICD implantation.

## Case presentation

We describe a case of a woman in her 40s with known and controlled ferropenic anemia and an anxiety disorder. The patient was on levonorgestrel (intrauterine route, 20 µg/24 hour), Ferrous sulfate (329.7 mg, once daily), and ethyl loflazepate (2 mg, as needed in acute anxious crises). The patient had no known allergies.

After visiting her father at their local hospital unit, the patient suddenly lost consciousness and started convulsing. She was immediately assisted by the emergency team. She was in cardiac arrest with ventricular fibrillation and was successfully defibrillated. Once the patient was stabilized after a successful return of spontaneous circulation (ROSC), she was evaluated by cardiology, was found to be in severe ventricular dysfunction, and was admitted to the intensive care unit for further monitoring and workup, which included an electrocardiogram, an echocardiogram, and blood tests.

The patient was then admitted to the cardiology department. After undergoing several exams, including a transthoracic echocardiogram, a coronary catheterization, and cardiac magnetic resonance imaging, she was diagnosed with Takotsubo cardiomyopathy (Broken heart syndrome) with indications to implant an ICD for secondary prevention against sudden death. The procedure took place without immediate complications, and the patient was started on bisoprolol (2.5 mg once daily) and lisinopril (5 mg once daily).

Six months after the above-mentioned episode, the patient presented to her primary care facility with complaints of both left-sided cervicalgia and left-arm brachialgia associated with left-arm edema. The patient stated that these symptoms started a couple of days after the implantation of the ICD. Slight left arm edema was noted on physical examination. Radial pulses were present bilaterally and broad. Upon palpation, left-sided cervical paraspinal muscle contractures and pain in the spinal apophysis were noted. No movement, passive or active, limitations were noticed, and the patient’s motor function was preserved. Jobe test, Patte test, lift-off test, palm-up test, and Yocum test were performed, none of which suggested a differential diagnosis for the major complaints of the patient. A cervical computerized tomography scan of the patient showed disc-osteophytic degenerative alterations but without any compromise of the myeloradicular space. The patient started left shoulder and cervical physiotherapy.

**Figure 1:**
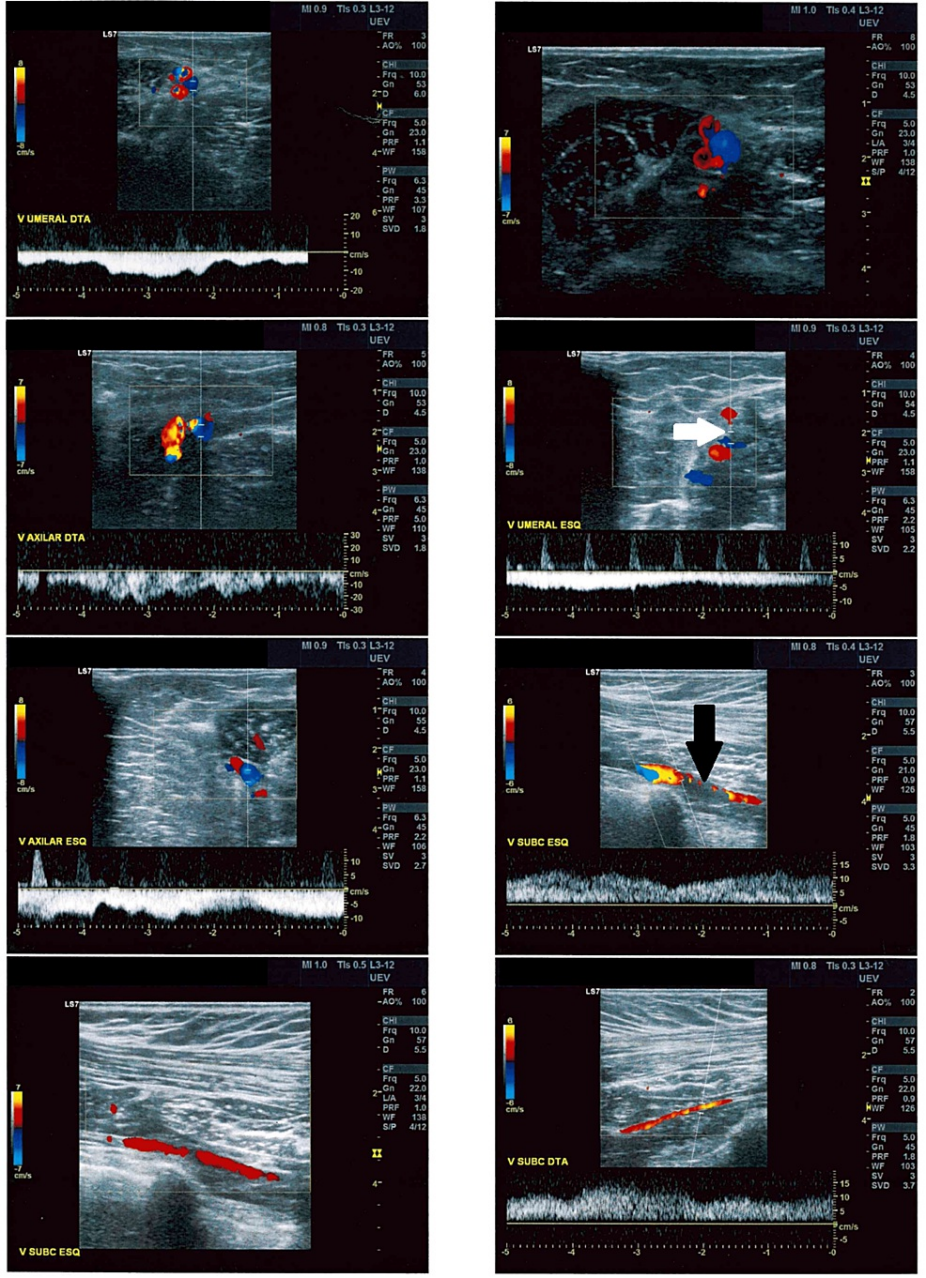
Doppler ultrasound images of the upper extremities Left humeral vein showing a reduction in the internal vein lumen (white arrow). Long-axis view of left subclavian vein thrombosis (black arrow).

One month later, the patient visited her family physician again because her complaints did not resolve despite the treatments prescribed. Upon physical examination, pain and edema were the most evident changes; therefore, a left-arm venous ultrasound with Doppler was done, which showed good permeability, good compressibility, absence of parietal and endoluminal lesions, valvular competence, phasic flow with respiratory movements, and distal compression maneuvers in the subclavian, axillary, humeral (proximal portion), radial, cubital, cephalic, and basilic veins. Despite these results, the exam showed pathological valvular reflux and parietal thickening (fibrosis) with a reduction of the internal lumen in the humeral vein in the middle and lower thirds of the arm, suggesting old DVT, which had been completely recanalized (Figure 1).

The patient was then referred to a vascular surgery specialist, who confirmed the diagnosis of DVT. She was started on an anticoagulant with apixaban (10 mg twice daily for the first week followed by 5 mg twice daily) and advised to use a compression sleeve. Three months later, the patient was asymptomatic and discontinued the prescribed medication regimen.

## Discussion

Upper extremity DVT is uncommon, representing 5-10% of all cases [7]. However, as previously mentioned, the increased use of devices such as ICDs or central venous catheters has made this diagnosis more common. Therefore, clinicians should have a more heightened index of suspicion.

In these cases, the most commonly affected veins are typically the more proximal ones as the brachial vein, whereas the more distal veins are affected less frequently [7]. Despite being rare, the relationship between ICD implantation and the occurrence of thromboembolism is already known. In particular, the three-month risk of DVT subsequent to ICD implantation stands at 0.3%, irrespective of the level of comorbidity. Meanwhile, the five-year risk increases to 1.9%, exhibiting more than a twofold elevation in patients with severe comorbidities compared to those without [6].

In a case report by Saroj-Mandal et al., 20 cases of DVT after ICD placement were analyzed, all regarding the subclavian vein [8]. In 45% of these cases, one of the manifestations was upper limb pain, as described in this case. Neck pain was not present among the signs and symptoms described at presentation, which included upper extremity swelling (90%), upper extremity erythema (15%), upper extremity venous claudication (10%), dyspnea (10%), and cough (10%). Only 30% of patients with this condition were female. The patients involved often had comorbidities, such as diabetes mellitus (45%), smoking (35%), hypertension (30%), obesity with body mass index ≥30 (30%), history of acute myocardial infarction (25%), chronic obstructive pulmonary disease (20%), and history of congestive cardiac failure (15%). Interestingly, our patient did not suffer from any known comorbidities.

The case we report is unique, as we did not find any case of DVT in the humeral vein after ICD implantation in the literature. The case is also peculiar because the condition presented with a set of nonspecific symptoms such as brachialgia and neck pain, which the patient undervalued. Regarding post-ICD-implantation DVT, our patient’s treatment was similar to what is recommended in the literature, as anticoagulation and early vascular consultation are both recommended [9]. Despite not being performed in this case, ICD lead extraction is another option in specific circumstances [10].

Venous thrombosis prophylaxis with hypocoagulable medications such as warfarin may be prescribed in some cases, especially in high-risk patients, to reduce these complications [11]. This strategy should be taken after weighing the risks and benefits of each individual case, and was not adopted in this case. Some degree of venous lesions or venous obstructions still occur in 14-50% of patients after the placement of such devices [12,13].

## Conclusions

The case we report is unique in that we were unable to find cases of DVT in the brachial veins after ICD implantation in the literature. In addition, what makes this case even more relevant is the fact that it presented itself in a non-specific way, leading to a rather late diagnosis of the condition. The installation of these cases sometimes takes place in an insidious way, with a set of non-specific manifestations. Therefore, the physician must be particularly attentive to patients with a history of ICD placement, considering the occurrence of DVT in the upper limbs as a diagnostic hypothesis to consider.
